# A novel method to detect the early warning signal of COVID-19 transmission

**DOI:** 10.1186/s12879-022-07603-z

**Published:** 2022-07-18

**Authors:** Mingzhang Li, Shuo Ma, Zhengrong Liu

**Affiliations:** School of Mathematics, South China University of Technology, Guangzhou, 510640 China

**Keywords:** Coronavirus disease 2019 (COVID-19), Early warning signals (EWS), Landscape network entropy (LNE), Auto-reservoir neural network (ARNN)

## Abstract

**Background:**

Infectious illness outbreaks, particularly the corona-virus disease 2019 (COVID-19) pandemics in recent years, have wreaked havoc on human society, and the growing number of infected patients has put a strain on medical facilities. It’s necessary to forecast early warning signals of potential outbreaks of COVID-19, which would facilitate the health ministry to take some suitable control measures timely to prevent or slow the spread of COVID-19. However, since the intricacy of COVID-19 transmission, which connects biological and social systems, it is a difficult task to predict outbreaks of COVID-19 epidemics timely.

**Results:**

In this work, we developed a new model-free approach, called, the landscape network entropy based on Auto-Reservoir Neural Network (ARNN-LNE), for quantitative analysis of COVID-19 propagation, by mining dynamic information from regional networks and short-term high-dimensional time-series data. Through this approach, we successfully identified the early warning signals in six nations or areas based on historical data of COVID-19 infections.

**Conclusion:**

Based on the newly published data on new COVID-19 disease, the ARNN-LNE method can give early warning signals for the outbreak of COVID-19. It’s worth noting that ARNN-LNE only relies on small samples data. Thus, it has great application potential for monitoring outbreaks of infectious diseases.

**Supplementary Information:**

The online version contains supplementary material available at 10.1186/s12879-022-07603-z.

## Background

Infectious illness outbreaks, particularly the recent pandemic of the coronavirus disease 2019, which continue to affect humanity, have posed enormous challenges to socio-economic progress. This dangerous infectious disease [1], whose mortality rate is very high, has brought a serious threat to human health. Various clinical trials and investigations [2–4] have shown that the COVID-19 may cause severe damage to the kidneys, liver, heart, and almost all organ systems in humans. Even after recovery, it can bring serious sequelae, including long-term negative effects on the nervous system, mental health, and the human body metabolism. Additionally, the global outbreak of COVID-19, resulting in absenteeism, indirectly caused incalculable economic losses, completely disrupted the world’s social and economic order [5]. Although some COVID-19 vaccines have been developed, humans still have to combat COVID-19 owning to the mutation of this dangerous virus.

There are numerous researches [6] demonstrate that pre-outbreak measures, such as social isolation and vaccine development, can contain the outbreak of infectious diseases. However, the cost of developing new infectious disease surveillance systems may be prohibitive for most developing countries [7, 8]. Lack of effective surveillance or adequate response could enable the emergence of new epidemic or pandemic patterns [9] from an endemic infection of SARS-CoV-2. From a public health and economic perspective, if an early warning signal can be given before an outbreak, the health ministry can take measures in advance to block or slow the spread of infectious diseases to prevent a new coronavirus disease epidemic or at least reduce the scale of an epidemic outbreak. Consequently, numerous machine learning methods [10] have been used to predict the trend of the epidemic, and various statistical models [11] are also utilized to analyze the spread of COVID-19. Nevertheless, predicting the outbreak of infectious diseases in real-time is still a challenge since coronavirus diseases are affected by many factors in the biological system and social system. Alternatively, the traditional machine learning method is difficult to deal with short-time high-dimensional data, and the deep learning method also needs lots of data. All these methods are easy to encounter the problem of over-fitting. Hence, it is of great significance to develop a novel approach for early warning of the outbreak of COVID-19.

**Fig. 1:**
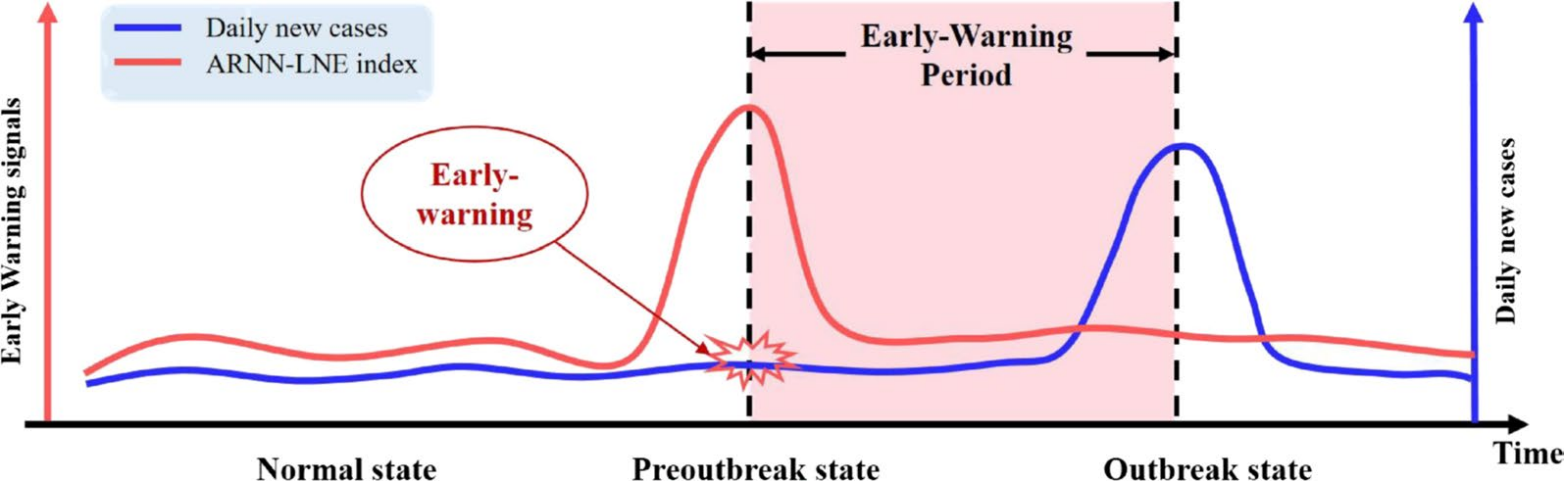
Early-Warning. The normal stage, the pre-outbreak stage, and the outbreak stage are the three stages of the COVID-19 dissemination process, according to the DNM theory. The rapidly increasing ARNN-LNE index, shown on the red curve, indicates a transition from the normal stage to the pre-outbreak stage, i.e., the critical point before the upcoming outbreak of COVID-19, as shown by the blue curve

To develop early warning methods, we can make simple assumptions that the transmission of an epidemic can be divided into three stages [12–14]: the normal stage, the pre-outbreak stage, and the outbreak stage, as shown in Fig. 1. The spread of Coronavirus Disease 2019 can be regarded as the dynamic behavior of the dynamical system, the critical transition of COVID-19 corresponds to the bifurcation of this dynamical system [12]. The principle for detecting the critical transition in this paper is based on the theory of dynamic network marker or biomarker [15] (DNM or DNB), by mining dynamic information from high-dimensional historical data. The DNM theory is a generalized method for identifying the critical transition before a catastrophic event. This method has been applied to many biological processes with remarkable results, including identifying the critical points of cellular differentiation [16], detecting the critical periods of various biological processes [17], and predicting the tipping points of infectious disease outbreaks [14]. Since information entropy [18] is a method to measure the uncertainty of the system, it can be utilized combined with DNM theory to derive a quantitative index, for measuring or detecting the state of the transmission process of the COVID-19 disease.

Recently, a short time series forecasting method [19] proposed by Chen et al., namely, Auto-Reservoir Neural Network (ARNN), to achieve accurate predicting future multi-step information. Based on the theory of DNM and ARNN, we recently proposed a new scientific method, called the network landscape entropy based on Auto-Reservoir Neural Network (ARNN-LNE). The algorithm can be described as follows. Firstly, a regional network [12] can be constructed to correlate the confirmed data of daily new cases in each region, where the daily new case data can be simply combined into high-dimensional short-term data. Secondly, the future information of these data can be predicted by the ARNN method [19]. Finally, a network entropy method [20] combined with the future information is used to obtain the critical early warning signal. The specific content of the ARNN-LNE method is described in “Methods”. Unlike the existing methods, this method can determine the COVID-19 contagion’s pre-outbreak stage, in which there is no obvious abnormality but a high risk of turning into an irreversible outbreak stage.

## Methods

### Auto-reservoir neural network

Many conventional forecasting algorithms have been used to predictability [9, 10] including autoregressive and autoregressive integrated moving average (ARIMA) and support vector regression (SVR). However, these approaches require sufficient training samples or data, such as high dimensional short-term time series or long-term time series, so it’s extremely difficult to predict the future evolution reliably only by using short-term time-series data. Theoretically, some neural network techniques including recurrent neural networks [21] (RNN) and long-term and short-term memory networks [22] (LSTM) could learn nonlinear dynamics from training data. But when there are a few samples are available for training networks, these algorithms encounter overfitting challenges frequently. Moreover, training neural networks might take a long time and require lots of computing resources.

**Fig. 2:**
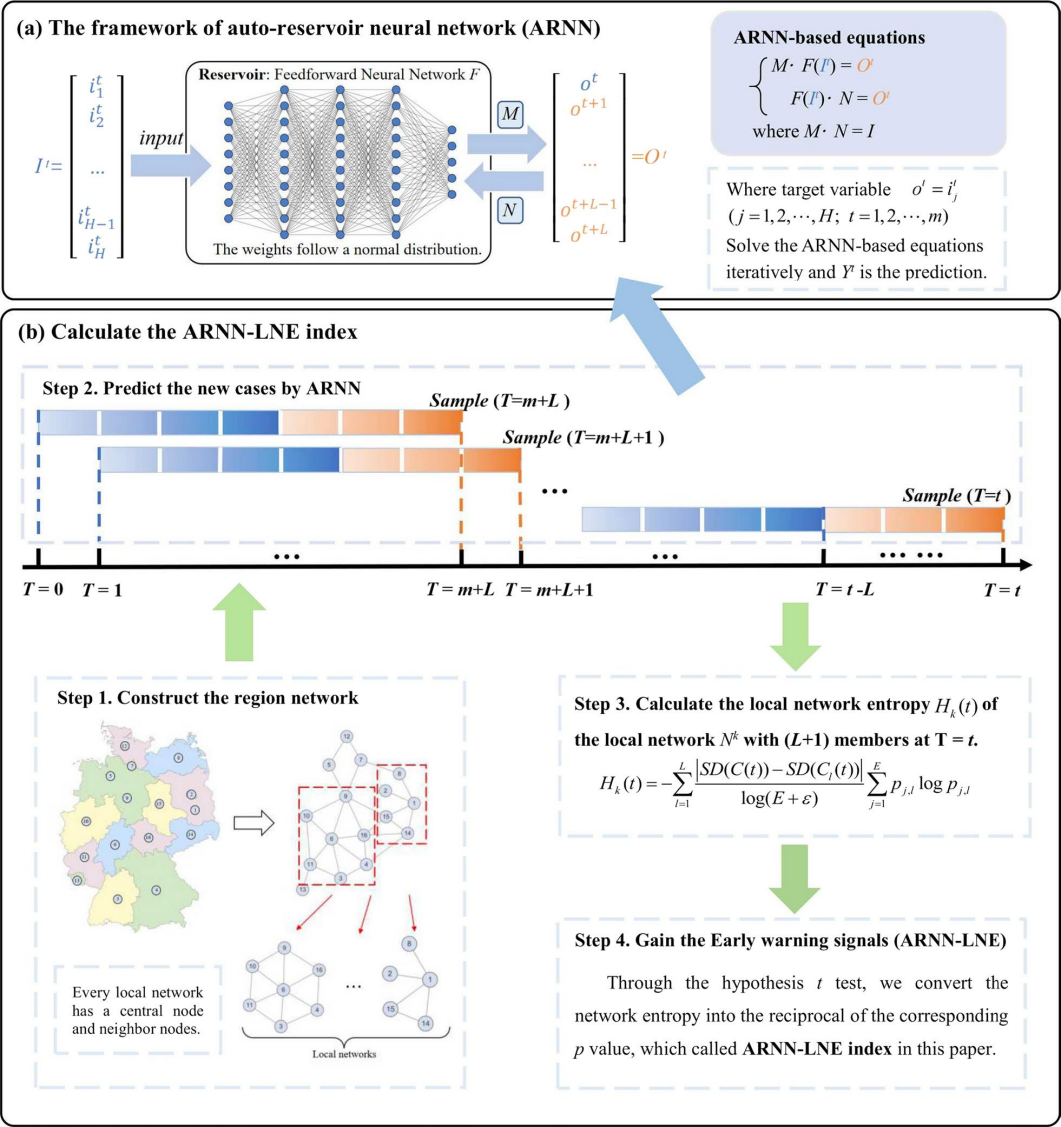
The main idea of ARNN-LNE. **a** The frame of the auto-reservoir neural network (ARNN). ARNN is a model-free multistep-ahead prediction approach for a target *y*. In the architecture of ARNN, the reservoir component consists of a fixed multilayer neural network *F* with a randomly assigned weight and data input $I_{t}$. Moreover, $O_{t}$ is a target vector generated by solving the ARNN-based equation iteratively. **b** Calculate the ARNN-LNE index. There are four steps to obtaining the early warning signals according to the ARNN-LNE approach. Step 1. Construct a regional network. Step 2. Predict daily new cases of COVID-19 by ARNN. The raw data were processed through window shift where window breadth is set as *m* + *L*. The blue part of the data is the training data and the orange part is the predicting data gained by ARNN method. Step 3. Calculate the local network entropy $H_{k} (t)$ of the local network $N_{k}$ with (*L* + 1) members at the time point $T = t$. Step 4. Gain the early warning signals (ARNN-LNE index)

To address these problems, a forecasting method called Auto-Reservoir Neural Network [19] (ARNN) was proposed. This network framework, as illustrated in Fig. 2a, translates the observed high-dimensional dynamic information into the reservoir and maps the high-dimensional spatial data to the target variable’s future time information. Specifically, assuming that there is an *H*-dimensional vector $I^{t} = (i_{1}^{t} ,i_{2}^{t} ,...,i_{H}^{t} )^{\prime}$ for each of $t = 1,2,...,m.$ A one-dimensional delayed vector $O^{t} = (o^{t} ,o^{t + 1} ,...,o^{m + L - 1} )^{\prime}$ matching to $I^{t}$ can be generated by the delay-embedding theory [23]. By combining reservoir computing (RC) [24] and the spatial–temporal information transformation (STI) [25, 26], an ARNN framework can be obtained, as shown in formula (1).

1 $$\left\{ \begin{gathered} MF(I^{t} ) = O^{t} \hfill \\ F(I^{t} ) = NO^{t} \hfill \\ \end{gathered} \right.$$

where $MN = I$, *M* is an $L \times H$ matrix and *N* is an $H \times L$ matrix and *I* represents an $L \times L$ identity matrix. The nonlinear function *F* in Eq. (1) can be provided by a multi-layer Feedforward neural network, which takes a hyperbolic tangent function $y = \tanh (x)$ as the activation function. The weights of the neural network *F* are random values that obey the Gauss distribution, so it’s not necessary to train the neural network. Through the ordinary least square method, we can solve the conjugate Eq. (1) iteratively, and obtain the future information of the target variable $(o^{t} ,o^{t + 1} ,...,o^{m + L - 1} )$ as well as the unknown weight matrices *M* and *N*. The prediction target variable *o* can be any of the high-dimensional observation variables, such as $o^{t} = i_{k}^{t} ,\;k = 1,2,...,H$. Moreover, *L* is the prediction step size, *H* is the number of observed variables, and *m* is the length of the observed data.

The daily new cases of COVID-19 for each region can be regarded as one-dimensional data and then the original data of multiple regions can constitute high-dimensional data, which contains important information about the dynamic system. Naturally, we could predict any region’s daily new cases by the ARNN method and the inputting data of ARNN is the high-dimensional data mentioned above.

### Dynamic network marker

The idea of dynamic network marker [12] (DNM) or dynamic network biomarker (DNB) is an elaboration of the critical slowing theory [27] of high-dimensional systems. We can employ the discrete dynamic system to express the dynamic development process of the regional network, provided that the spread of an infectious disease is a complex dynamic process of a nonlinear system. When a complex system approaches a critical point or tipping point, the DNM theory states that there exists a dominant group, i.e., the DNM Group, which fulfills three basic properties:

i. Within the DNM group, the Pearson correlation coefficient (PCC) between each pair of members rises significantly.
ii. The Pearson correlation coefficient (PCC) between the DNM Group member and the non-DNM Group member drops rapidly.
iii. For each member of the DNM group, the standard deviation (SD) increases dramatically.

The emergence of the DNM group with strong fluctuation and high correlation signifies the arrival of the critical transition, according to the properties given above. As a result, these traits can be utilized as three criteria to characterize a complex biological system’s critical state.

### The algorithm of ARNN-LNE

Based on ARNN and DNM methods, we propose a novel critical warning method for infectious diseases, namely, the landscape network entropy based on the auto-reservoir neural network (ARNN-LNE). The calculation process of this method is mainly divided into the following four steps, as shown in Fig. 2b.

- [Step 1]: Constructing a regional network structure

In a country or region, the geographical location information is modeled to a network, where each node represents a region. There is an edge between two adjacent areas in this network, indicating their adjacency relationship. Taking Germany as an example, based on the geographic locations and traffic routes of these 16 provinces, a regional network can be constructed as shown in Fig. 2b, which has 16 nodes and 27 edges. This network can also be partitioned into numerous local networks, which are composed of central nodes with their first-order neighbors. Therefore, a local network $N^{k}$ has *E* + 1 members, that is, a central node *k* with its first-order neighbor nodes $k_{j} \;\;(j = 1,2,...,E)$.

- [Step 2]: Predicting the daily new cases time series of COVID-19 by ARNN

The daily new data for each region can be regarded as one-dimensional data and then the original data of multiple regions can constitute high-dimensional data. For each time point $T = t$, choosing the appropriate training length *m* and prediction length *L*, we could use the high-dimensional data $I^{t}$ as the input and the future predictions $O^{t}$ can be obtained by solving the ARNN-STI equation iteratively, as depicted in Fig. 2b.

- [Step 3]: Calculate the ARNN-LNE index

For any local network $N^{k}$ with *E* + 1 members, its network entropy index $H_{k}$ at the time point $T = t$ can be calculated according to formulas (2), (3).

2 $$H_{k} (t) = - \sum\limits_{l = 1}^{L} {\frac{{\left| {{\text{SD}}(C^{k} (t)) - {\text{SD}}(C_{l}^{k} (t))} \right|}}{\log (E + \varepsilon )}\sum\limits_{j = 1}^{E} {p_{j,l}^{k} \log p_{j,l}^{k} } }$$

3 $$p_{j,l}^{k} = \frac{{\left| {{\text{PCC}}(C_{j}^{k} (t),C^{k} (t)) - {\text{PCC}}(C_{j,l}^{k} (t),C_{l}^{k} (t))} \right|}}{{\sum\limits_{j = 1}^{E} {\left| {{\text{PCC}}(C_{j}^{k} (t),C^{k} (t)) - {\text{PCC}}(C_{j,l}^{k} (t),C_{l}^{k} (t))} \right|} }}\;\;\left( {l = 1,...,L\;;\;j = 1,2,...,E} \right)$$

where $C^{k} (t) = \left( {c^{k} (t - L + 1),c^{k} (t - L + 2), \cdots ,c^{k} (t)} \right)$ represents the sequence of daily new cases in the local network or region $N^{k}$ at the time point $T = t$, $c^{k} (t)$ denotes the new confirmed cases of COVID-19 at $T = t$ and *L-*1 is the predicting length. While $C_{l}^{k} (t) = \left( {c^{k} (t - L + 2),...,c^{k} (t),\overline{c}^{k} (t + l)} \right)$ stands for the predicted sequence of daily new cases at $T = t$*.* Calculated by the ARNN method in [Step 2], $\overline{c}^{k} (t + l)$ is the predicted daily new cases in the region $N^{k}$ at $T = t + l$. In addition,$C_{j}^{k} (t)$,$C_{j,l}^{k} (t)$ in formulas (3) are the sequence and the predicted sequence of daily new cases in the first-order neighbor node $k_{j} \;\;(j = 1,2,...,E)$ of the central node *k* at $T = t$, respectively. According to the local network entropy $H_{k} (t)$, the average network entropy of the whole region $H_{t} = \sum\limits_{k = 1}^{K} {H_{k} }$ can be calculated. Additionally, the number of local network members considered here must be at least more than 2, that is, the number of neighbors of the central node *k* is greater than 1. If a center node has no neighbor node or only has one neighbor, we let $p_{j,l} = 1$ to guarantee the normal calculation of formulas (2), (3).

- [Step 4]: Identify the pre-outbreak stage

The landscape network entropy $H_{t}$ can quantitatively detect the warning signal of critical transition from the normal stage to the outbreak stage. Through the hypothesis *t*-test, we can convert $H_{t}$ into the reciprocal of the corresponding *p*-value, which is called the ARNN-LNE index in this paper. When $p < 0.05$, we can see $H_{t}$ to be significantly different from the mean value of the vector $(H_{1} ,H_{2} ,...,H_{t - 1} )$, the time point $T = t$ can be regarded as the tipping point of the epidemic. Hence, the threshold for the ARNN-LNE index is set at 20, corresponding to the significance level $p = 0.05$. If the ARNN-LNE indicator is lower than the threshold, the state of the infectious disease is considered to be in the normal stage at the time point $T = t$, and then the new calculation will continue at the next time point $T = t + 1$. When the ARNN-LNE indicator exceeds the threshold, it can be regarded as a formal early warning signal.

From the perspective of a complex system, the dynamic process of the spread of COVID-19 can be described by the evolution process of a nonlinear dynamic system with bifurcation points, where the system undergoes drastic changes. The ARNN-LNE method is designed to detect the pre-outbreak stage before the catastrophic transition to the outbreak stage and is applied to six countries or regions. Specific experimental results in “Results” for analysis.

### Data processing and the parameter in ARNN-LNE

In this paper, the algorithm is applied to COVID-19 epidemic datasets [33, 34] in six countries or regions, including Germany, Italy, Netherlands, Spain, parts of Europe, and Canada. Considering that data collection may generate noise, we perform moving average processing on the acquired original data to reduce the impact of noise. The moving average lengths applied to each dataset are shown in Table 1. In addition, if the raw data of COVID-19 are less than or equal to 0, it would be replaced by the average data of the previous 3 days.

**Table 1:** The moving average length setting and the parameter settings in ARNN-LNE Algorithm

Country/Area	Germany	Spain	Canada	Italy	Netherlands	Parts of Europe
Moving average length	4	3	3	5	3	5
Train Length (*m*)	14	12	10	16	11	24
Predicting Length (*L*-1)	4	4	4	5	4	5

As shown in Fig. 2a, the ARNN framework directly converts the observed high-dimensional dynamic information $I^{t} = (i_{1}^{t} ,i_{2}^{t} ,...,i_{D}^{t} )^{\prime},\quad t = 1,2,...,m$ into the reservoir, and maps the high-dimensional spatial data to a one-dimensional delay time vector $O^{t} = (o^{t} ,o^{t + 1} ,...,o^{t + L - 1} )^{\prime}$, where *m* is the training length and *L*-1 is the predicting length. The parameters including *m* and *L* applied to the six datasets are shown in Table 1.

## Results

As a model-free method of nonlinear event prediction, the ARNN-LNE method has been applied to the datasets of COVID-19 confirmed cases from six nations or regions, including Germany, Canada, Italy, Netherlands, Spain, and parts of Europe. The time ranges of datasets are exhibited in the Table 2 and the ARNN-LNE method’s parameters for each dataset are listed in Table 1.

**Table 2:** The time ranges of the COVID-19 datasets

Country or Region	Time ranges of the datasets
Germany	2021/06/28–2021/09/29
Canada	2021/06/16–2021/10/17
Italy	2021/06/10–2021/10/12
Netherlands	2021/09/15–2021/12/19
Spain	2021/05/13–2021/10/12
Parts of Europe	2020/07/20–2021/10/12

### The application of ARNN-LNE in several countries

**Fig. 3:**
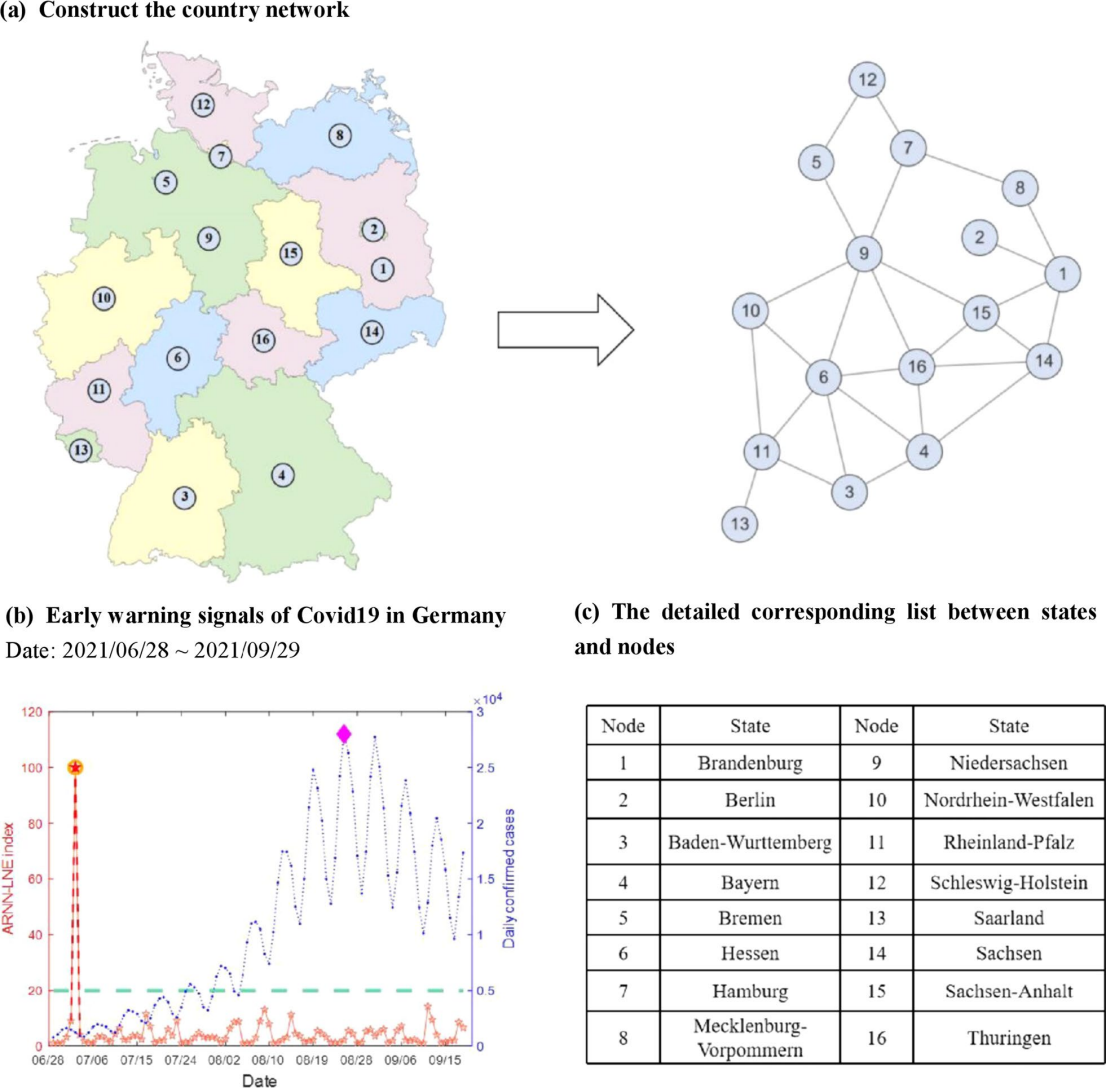
The early warning signals of COVID-19 in Germany. **a** Construct the country network by German geographical location. **b** The early warning signal of the outbreak of COVID-19 in Germany between June 28, 2021 and September 29, 2021. The red line depicts the ARNN-LNE index calculated by the algorithm in Sect. 2.2, whereas the blue line represents the daily news case of COVID-19, which was derived from raw data after smoothing filtering processing. The ARNN-LNE index can be considered an early warning signal if it exceeds this green line which stands for the threshold. The yellow circle with a red star is the formal signal, while the purple diamond denotes the peak of new daily cases. **c** The detailed information about the nodes in this network

In this work, we collected the daily new cases [33] of the COVID-19 epidemic of 16 German provinces from June 28, 2021 to September 29, 2021. As depicted in Fig. 3a, we can construct a regional network with 16 nodes and 27 edges based on the adjacency information for German geographical location. The detailed information of each node can be seen from Fig. 3c. A yellow warning signal given on July 3, 2021, as can be seen from Fig. 3b, indicates that the COVID-19 epidemic would enter the outbreak stage. As the highly transmissible variant (Delta) of SARS-CoV-2 swept all over the world, the number of confirmed cases began to increase in early July. Subsequently, the German disease control agency RKI assessed [28] that Germany entered the outbreak stage on August 20, 2021. Obviously, the date of the early warning signal provided by ARNN-LNE is earlier than RKI's warning signal.

**Fig. 4:**
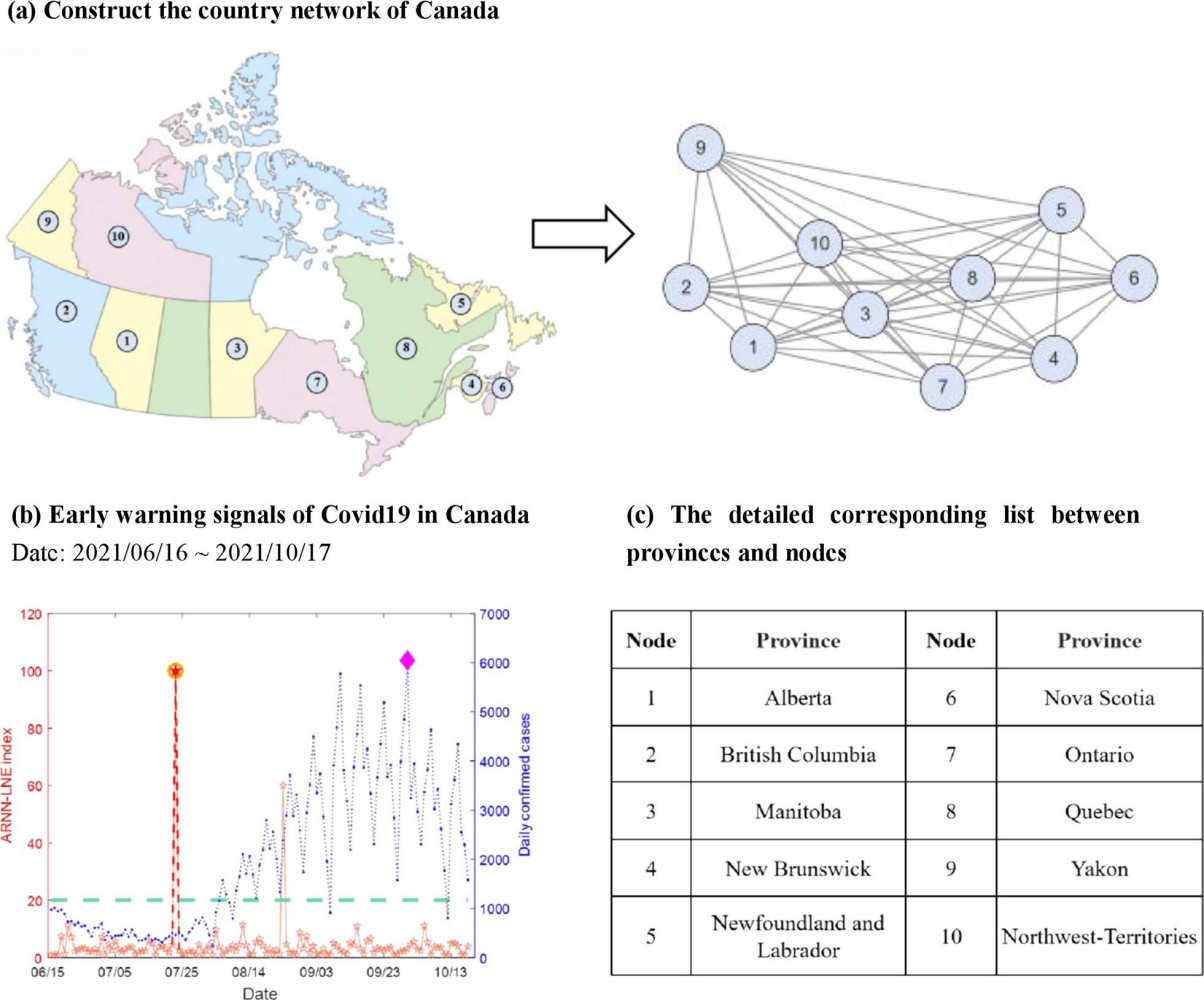
The early signals of COVID-19 in Canada. **a** Construct the country network by traffic information. **b** The early warning signal of the outbreak of COVID-19 in Canada between June 16, 2021 and October 17, 2021.The red line depicts the ARNN-LNE index calculated by the algorithm in “Methods”, whereas the blue line represents the daily news case of COVID-19, which was derived from raw data after smoothing filtering processing. The ARNN-LNE index can be considered an early warning signal if it exceeds this green line which stands for the threshold. The yellow circles are the formal signals, while the purple diamond denotes the local peak of daily new cases. **c** The detailed information about the nodes in this network

For Canada’s 10 provinces, we gathered daily data [34] of the COVID-19 epidemic from June 16, 2021 to October 17, 2021. In that Canada has a very large area and inter-provincial transportation is mainly aviation flight, we constructed a fully-connected regional network with 10 nodes, as illustrated in Fig. 4a. In this way, any two nodes are connected by an edge, and the corresponding region of each node is listed in Fig. 4c. At the end of 2021, the number of new cases per day began to increase rapidly as the government eliminated the remaining public health measures. An early warning signal, as presented in Fig. 4b, was provided by ARNN-LNE method on July 22, 2021, indicating that an outbreak of the COVID-19 is imminent. Canada’s chief public health officer, Theresa Tam, issued a warning [29] at a press conference on August 12, 2021, declaring that an epidemic was emerging in Canada and cases were developed along a strong recovery trajectory. Evidently, the time point of the ARNN-LNE warning signal is earlier than the warning date issued by the government.

**Fig. 5:**
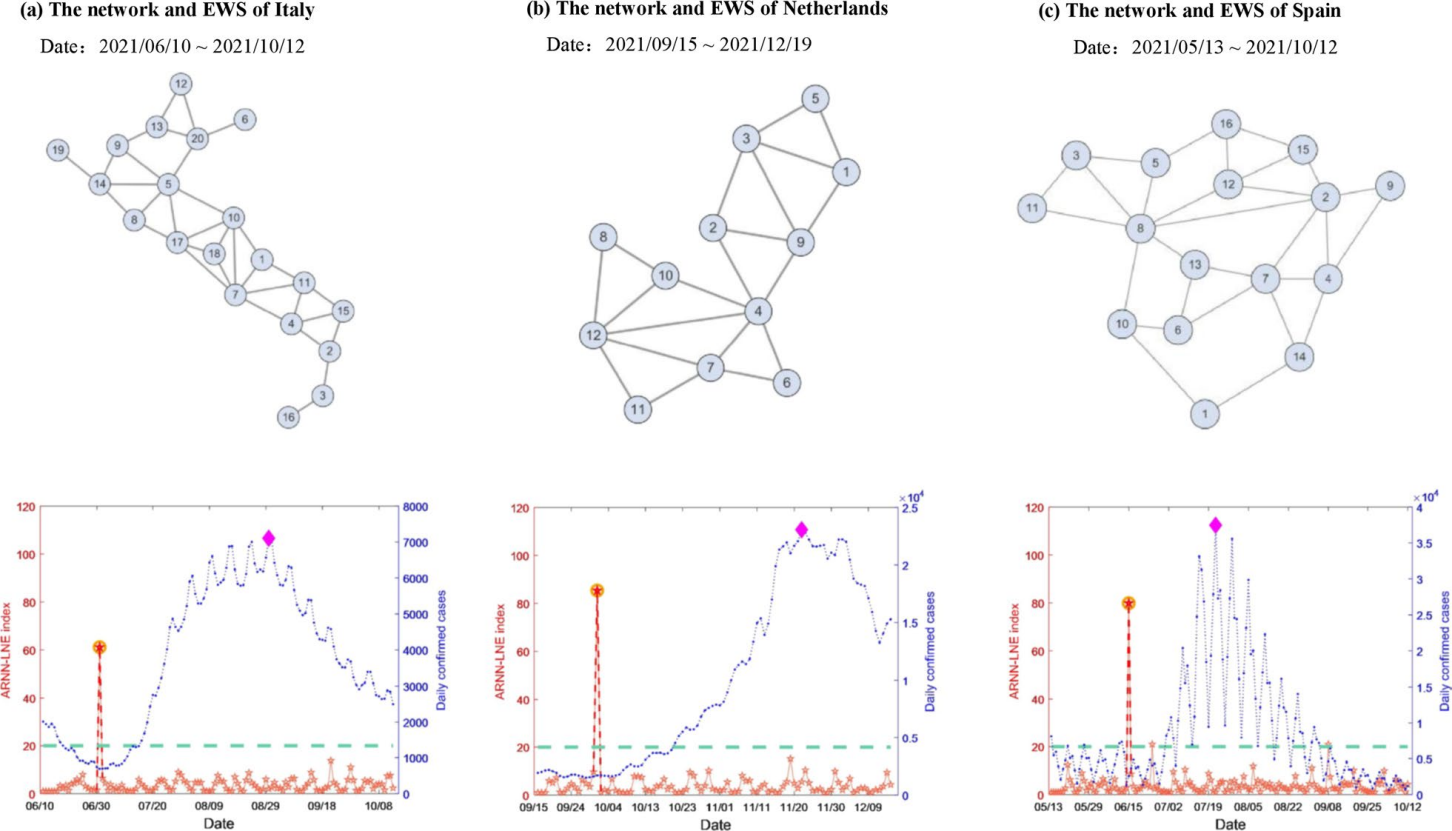
The early warning signals (EWS) of COVID-19 in Italy, Netherlands and Spain. There are three countries’ results of early warning given by the ARNN-LNE method. The regional networks are placed in the upper of the Figure and the results of ARNN-LNE index locates in the lower

In addition, the ARNN-LNE method is also applied to the historical datasets [33] of Italy, Netherlands, and Spain. For Italy, an early warning signal occurred on June 13, 2021, as portrayed in Fig. 5a. The epidemic in Italy entered the outbreak stage in early July, with a rapid increase in new confirmed cases per day. For Netherlands and Spain, the signals provided by ARNN-LNE all emerged before the dramatic rise of the new case series, as demonstrated in Fig. 5b, c. These signals are also supported by government-issued emergency events. See the Additional file 1: Figures for details.

### The application of ARNN-LNE in parts of Europe

**Fig. 6:**
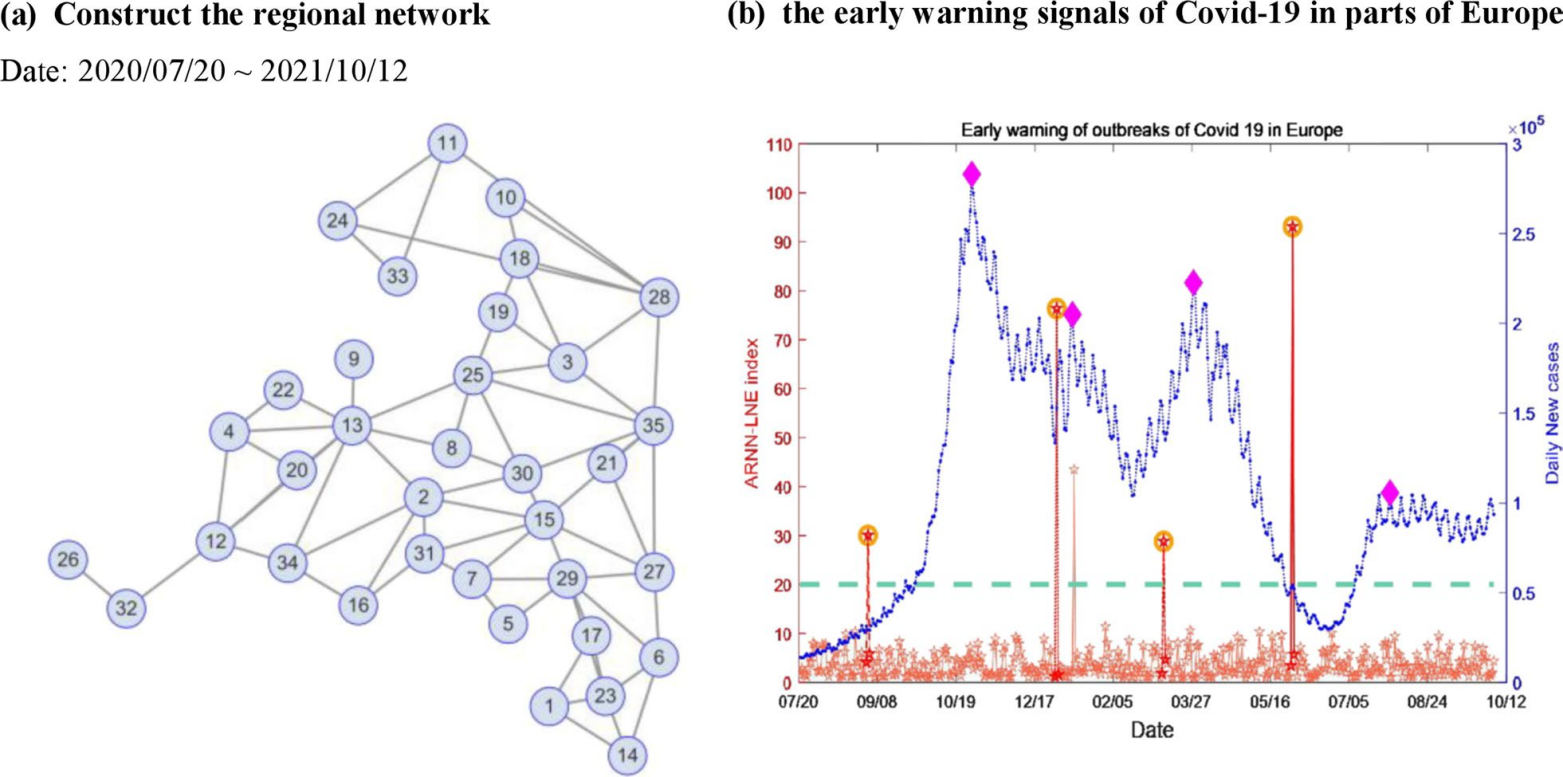
The early signals of COVID-19 in parts of Europe between July 20, 2020 and October 12, 2021. **a** Construct the regional network by geographical location of parts of Europe. **b** The Early warning of outbreak of COVID-19 in parts of Europe

Not limited to the analysis of COVID-19 in a single nation, we also acquired historical data [33] on daily new COVID-19 cases from July 20, 2020 to October 12, 2021 in 35 European countries. As shown in Fig. 6a, a regional network can be constructed based on geographical location. This network has 35 nodes, which represent one country, and 69 edges. See the Additional file 1: Fig. S4 for details. An early warning signal, as can be seen from Fig. 6b, was received by the ARNN-LNE method on September 1, 2020, indicating that the COVID-19 epidemic in Europe will enter the outbreak stage. Between July and October, the number of COVID-19 cases increased at an exponential rate, peaking in the first half of November. Europe became the epicenter of the pandemic at the end of 2020, despite the deployment of the COVID-19 vaccine in numerous countries. As a result, every country in Europe has to take some tougher measures to prevent the spread of COVID-19. On March 9, 2021, ARNN-LNE gave the early warning signal, which indicates that the epidemic started to enter the outbreak stage again, as depicted in Fig. 6b. Due to the mutation of the new COVID-19 and the stagnation of vaccination programs, the number of new confirmed cases each day has increased rapidly, reaching a local peak again in late April, and European countries such as Germany, Italy have also entered a new round of blockade. On May 29, 2021, an early warning signal was provided by ARNN-LNE, although the real confirmed cases were still at a low level. However, the number of daily new confirmed cases of COVID-19 throughout Europe had risen dramatically and the outbreak of the COVID-19 epidemic had reappeared in the last two weeks. In general, the ARNN-LNE method can provide early warning signals before infectious disease outbreaks.

## Discussions

Although the vaccine for the COVID-19 epidemic has been developed and is now available in all countries, the situation of this epidemic is unlikely to contain rapidly. To reduce the risk of COVID-19 infection in humans and alleviate the shortage of medical supplies, we need to present scientific approaches for the relevant medical departments to execute appropriate control measures promptly. The ARNN-LNE method, which has been proposed in this paper, is a novel way for early warning of infectious disease outbreaks. This method utilizes short-time-series samples to obtain early warning signals and has great potential for real-time surveillance of emerging COVID-19 infectious illnesses, as evidenced by its successful implementation in six countries or regions.

In addition, ARNN-LNE is a model-free scientific calculation method, which is not directly related to the mechanism of infectious disease transmission. But the change of ARNN-LNE’s warning signal should correspond to the change of the basic regeneration number *R*_0_ [30], which is an indicator of describing the likelihood of infectious organisms spreading in a population not previously immunized. Theoretically, $R_{0} = 1$ corresponds to the bifurcation point of the nonlinear dynamical system [31] of COVID-19. Table 3 lists certain countries’ early warning signals of COVID-19, as well as their *R*_0_ information [32].When the ARNN-LNE index exceed 20, it can be an early warning signal. Evidently, *R*_0_ was near to 1 at the time point when the ARNN-LNE index is provided, indicating that the proposed ARNN-LNE method can provide an early warning signal of a disease outbreak before a critical transition from a normal state to an outbreak state of infectious disease.

**Table 3:** The detailed information of ARNN-LNE index and R0

Country	Date	ARNN-LNE index	R0
Germany	2021/07/03	100	0.85
Canada	2021/07/22	100	1.04
Spain	2021/06/13	79.8	1.03
Netherlands	2021/10/01	85.4	0.99
Italy	2021/06/30	61.1	0.81

**Fig. 7:**
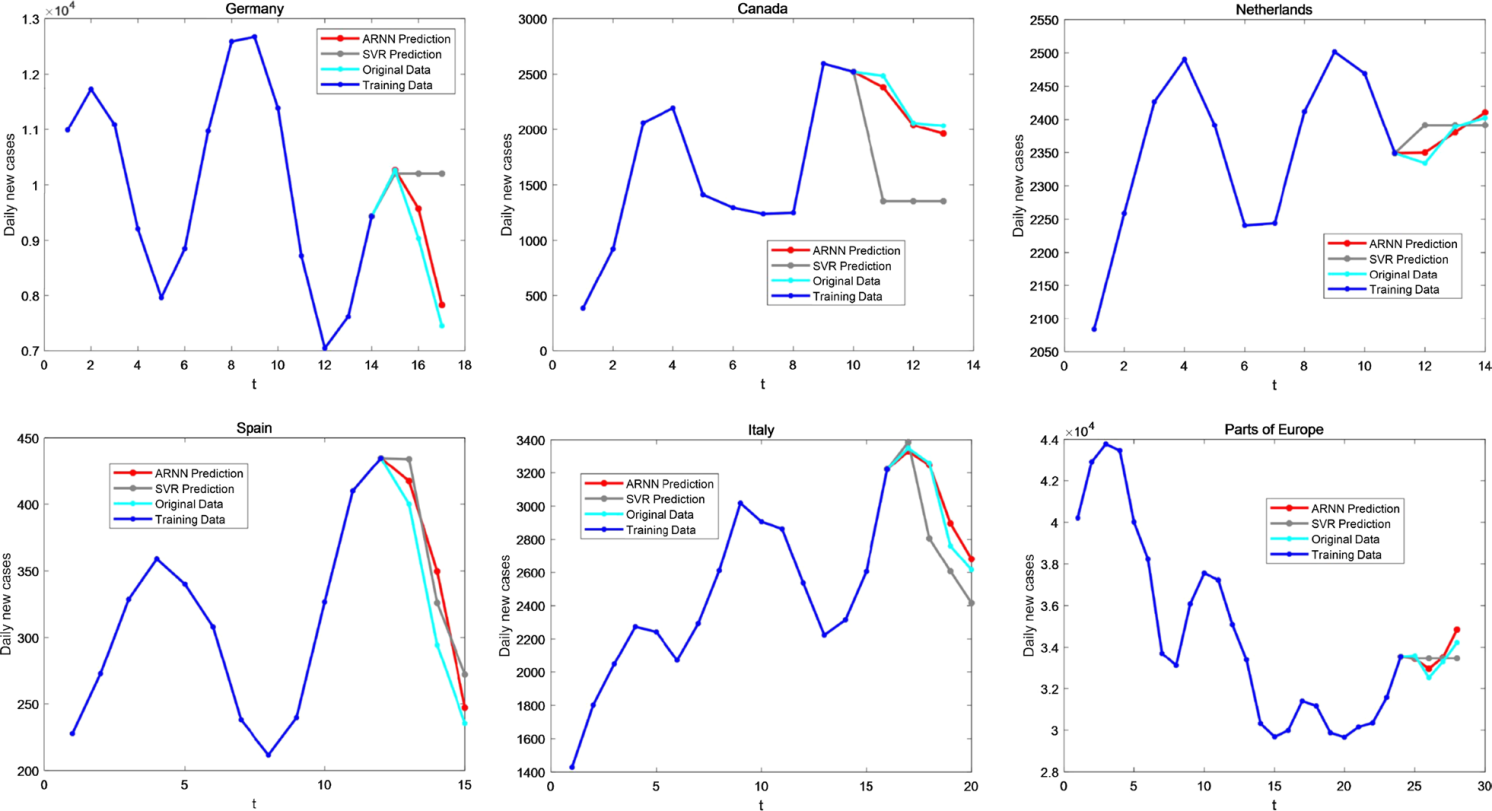
Comparison of prediction results between ARNN and SVR. We compared our method with the traditional machine learning method SVR in predicting the new COVID-19 epidemic based on the following data-sets of COVID-19 confirmed case: Germany, Canada, Netherlands, Spain, Italy and Europe. The blue line represents the training data of COVID-19 daily new case and blue green line represents the original data of COVID-19. The gray line is the prediction obtained by using SVR method, while the red line is the prediction of COVID-19 obtained by using ARNN

To test the accuracy of ARNN-LNE, we compared our method with the traditional model in two ways. On the one hand, we compared ARNN with the traditional machine learning method SVR in predicting the daily new COVID-19 cases based on the six data-sets mentioned in the paper. The results of the comparison are shown in Fig. 7 and Table 4, which indicate that ARNN outperforms SVR in prediction.

**Table 4:** The root-mean-square error (RMSE) of SVR prediction and ARNN prediction

Region Method	Canada	Germany	Italy	Netherlands	Spain	Parts of Europe
SVR	759.812	1778.514	116.317	115.6	15.29	4416.937
ARNN	35.117	160.372	34.15	5.371	15.26	151.178

**Fig. 8:**
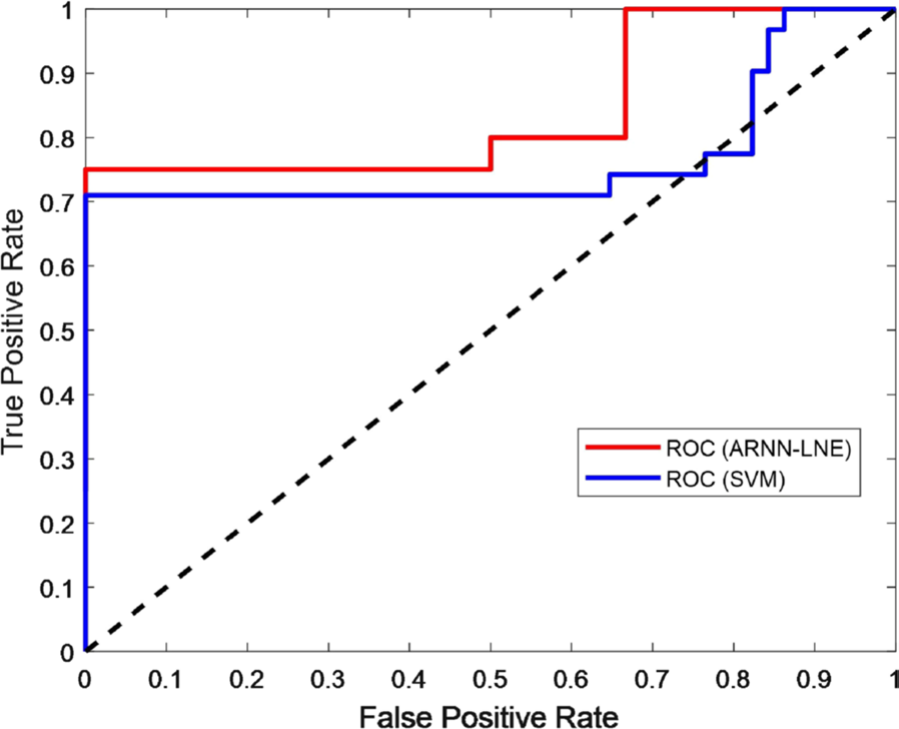
The performance comparisons of ARNN-LNE and support vector machine (SVM)

One the other hand, regarding the early warning of the COVID-19 epidemic as a binary classification problem, that is to distinguish whether the current state of the dynamic system is in a critical state or a stable state, we compared the proposed ARNN-LNE method with the traditional machine learning model, support vector machine (SVM). As can be seen clearly in Fig. 8, the performance of the ARNN-LNE method is better than the SVM-based system. It’s easy to calculate that the AUC of ARNN-LNE is 0.825 and the AUC of SVM is 0.77.

Compared with traditional machine learning algorithms, the ARNN-LNE method has the following advantages. Firstly, ARNN-LNE is a model-free approach that does not require training or testing procedures and feature selection during computation. Further, because the method is data-driven and has no direct relationship with the mechanism of epidemic spread, it can also be applied to other infectious diseases besides COVID-19 such as hand-foot-and-mouth disease. Secondly, ARNN-LNE can rely on small samples rather than long-term time-series data. Therefore, it is suitable for application in some developing countries which lack public health infrastructure. Thirdly, ARNN-LNE is performed according to the predictive information. Thus, it would give warning signals earlier than conventional methods.

Our proposed method is a data-driven approach without modeling the dynamics of the transmission of infectious diseases. In fact, numerous studies [35–42] have shown that proactive measures taken by governments to deal with the outbreaks are beneficial to control the spread of infectious diseases. Timely measures quickly taken by the government before the outbreak could lead to changes in early warning signals, which we did not take into account. In future work, we will try to analyze the changes in early warning signals under the active control of the government, and attempt to make further improvements to our algorithm.

## Conclusions

In this paper, we proposed a model-free early warning of the epidemic method, i.e., ARNN-LNE. Based on the published data of the daily new COVID-19 cases, this approach can provide early warning signals for the outbreak of COVID-19. Specifically, ARNN-LNE can utilize the prediction information of time series to make an early warning, which performs better than some traditional machine learning models. To verify the effectiveness of the ARNN-LNE algorithm, we selected six nations or regions for critical transition warnings. The results of these numerical experiments prove that the proposed algorithm is valid and flexible. It’s worth noting that ARNN-LNE only relies on small sample data, rather than long-term data. Therefore, it has great application potential for monitoring outbreaks of infectious diseases. In the future, the COVID-19 epidemic will still bring serious harm to human society. Thus, it is very crucial to detect real-time changes and send out accurate warning signals of the COVID-19 outbreaks. We hope that our work can provide a reference for health institutions.

## Supplementary Information

**Additional file 1: Figure S1.** The country network of Spain. **Figure S2**. The country network of Italy. **Figure S3.** The country network of Netherlands. **Figure S4.** The regional network of Parts of Europe. **Figure S5.** The early signals of COVID-19 in Netherlands and Spain.

## Data Availability

The historical raw datasets on COVID-19 in Germany, Italy, Netherlands, Spain, and parts of Europe are available in the [JHU CSSE COVID-19 Data] repository, [https://github.com/cssegisanddata/COVID-19]. The historical original data in Canada is available from the Dalla Lana School of Public Health, University of Toronto, [CovidTimelineCanada] repository, [https://art-bd.shinyapps.io/covid19canada/].

## Abbreviations

COVID-19: Coronavirus Disease 2019
LNE: Landscape network entropy
ARNN: Auto-reservoir neural network
ARNN-LNE: The landscape network entropy based on Auto-Reservoir Neural Network
DNB/DNM: Dynamic network biomarker/maker
ARIMA: Autoregressive and autoregressive integrated moving average
SVR: Support vector regression
SVM: Support vector machine
LSTM: Long short-term memory
RNN: Recurrent neural networks
AUC: Area under the curve
EWS: Early warning signals
