# Supplementary material for: A novel method to detect the early warning signal of COVID-19 transmission
Source: BMC Infect Dis. 2022 Jul 18;22:626. doi: 10.1186/s12879-022-07603-z (PMC9289935; doi:10.1186/s12879-022-07603-z)
Supplement: Supplementary file 1 — Additional file 1: Figure S1. The country network of Spain. Figure S2. The country network of Italy. Figure S3. The country network of Netherlands. Figure S4. The regional network of Parts of Europe. Figure S5. The early signals of COVID-19 in Netherlands and Spain. [file 12879_2022_7603_MOESM1_ESM.pdf]

Supporting information of a novel method to detect the early warning signal of Covid-19 transmission

Mingzhang Li, Shuo Ma and Zhengrong Liu

Supplementary Figures

Node	State	Node	State
1	Andalusia	9	Catalonia
2	Aragon	10	Extremadura
3	Asturias	11	Catalonia
4	Valencia	12	La Rioja
5	Cantabria	13	Madrid
6	Castilla-La	14	Murcia
7	Mancha	15	Navarre

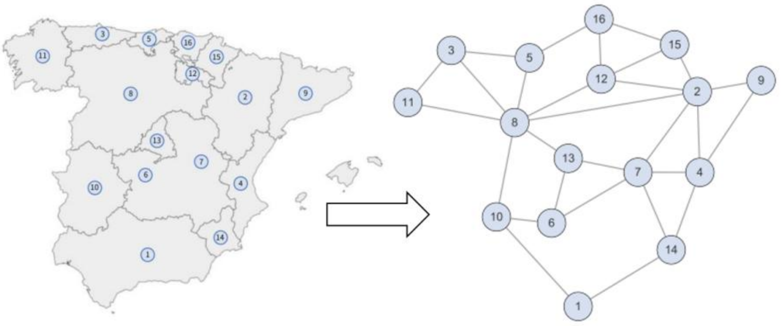

Figure S1: The country network of Spain. Based on the adjacency information of the geographic location, we can construct a regional network of Spain with 15 nodes and 28 edges. Each node represents a province or state and the edge represents the geographical adjacency between the two nodes or states.The detailed list of correspondences between regions/autonomous provinces and nodes is shown in Figure S1.

Node	State	State	Node
1	Abruzzo	11	Molise
2	Basilicata	12	P.A.Bolzano
3	Calabria	13	P.A.Trento
4	Campania	14	Piemonte
5	Emilia Romagna	15	Puglia
6	Friulli Venezia Giulia	16	Sicilia
7	Lazio	17	Toscana
8	Liguria	18	Umbria
9	Lombardia	19	Valle d'Aosta
10	Marche	20	Veneto

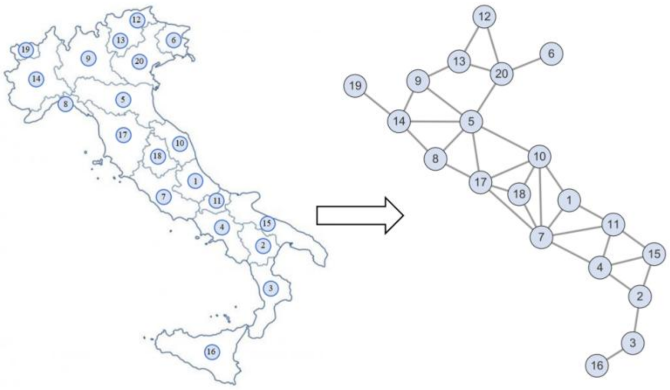

Figure S2: The country network of Italy. Based on the adjacency information of the geographic location, we can construct a regional network of Italy with 21 nodes and 34 edges. Each node represents a province or state and the edge represents the geographical adjacency between the two nodes or states. The detailed list of correspondences between regions/autonomous provinces and nodes is shown in Figure S2.

Node	State	Node	State
1	Drenthe	7	NorthBrabant
2	Flevoland	8	NorthHolland
3	Friesland	9	Overijssel
4	Gelderlan d	10	utrecht
5	Groninge n	11	Zealand
6	Limburg	12	South Holland

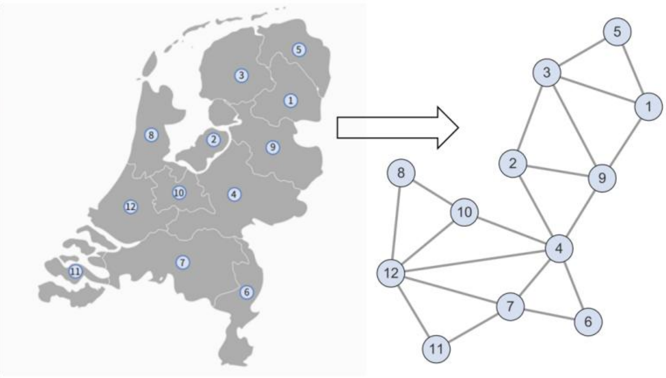

Figure S3: The country network of Netherlands. Based on the adjacency information of the geographic location, we can construct a regional network of Netherlands with 12 nodes and 20 edges. Each node represents a province or state and the edge represents the geographical adjacency between the two nodes or states. The

detailed list of correspondences between regions/autonomous provinces and nodes is shown in Figure S3.

Node	Country	Node	Country
1	Albania	19	Lithuania
2	Austria	20	Luxembourg
3	Belarus	21	Moldova
4	Belgium	22	Netherlands
5	Bosnia and Herzegovina	23	Macedonia
6	Bulgaria	24	Norway
7	Croatia	25	Poland
8	The Czech Republic	26	Portugal
9	Denmark	27	Romania
10	Estonia	28	Russia
11	Finland	29	Serbia
12	France	30	Slovakia
13	Germany	31	Slovenia
14	Greece	32	Spain
15	Hungary	33	Sweden
16	Italy	34	Switzerland
17	Kosovo	35	Ukraine
18	Latvia		

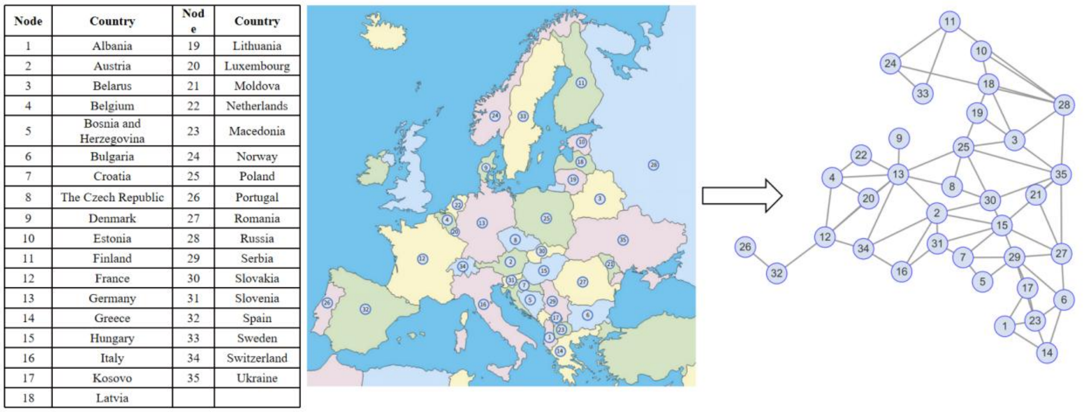

Figure S4: The regional network of Parts of Europe. Based on the adjacency information of the geographic location, we can construct a regional network of Parts of Europe with 35 nodes and 69 edges. Each node represents a country and the edge represents the geographical adjacency between the two nodes (or countries).The detailed list of correspondences between regions/autonomous provinces and nodes is shown in Figure S4.

(a) The country network and the early warning signals of Netherlands

Date：2021/09/15 ~ 2021/12/19

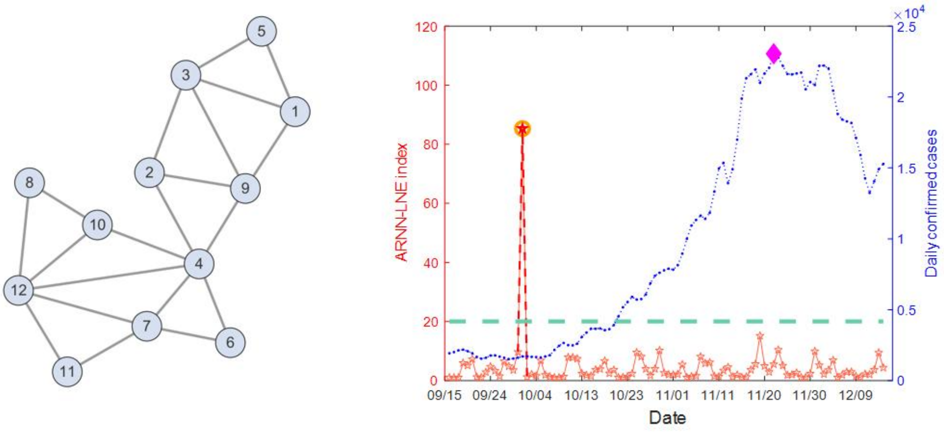

(b) The country network and the early warning signals of Spain

Date：2021/05/13 ~ 2021/10/12

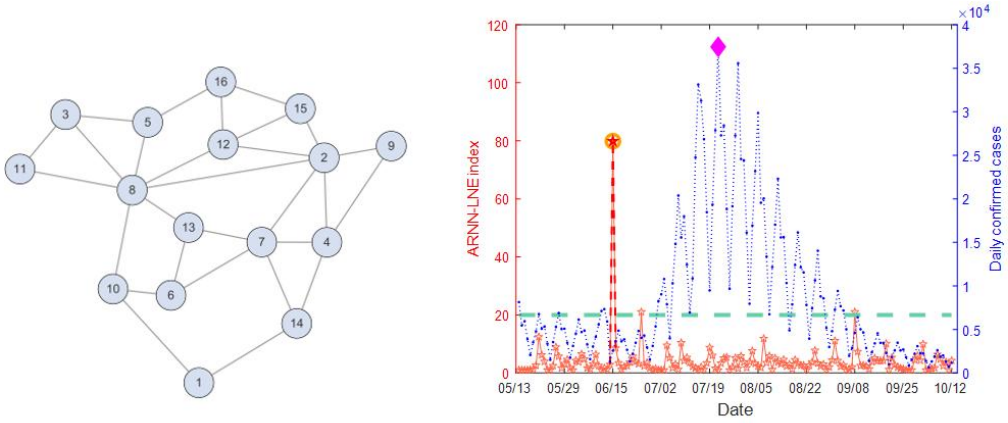

Figure S5: The early signals of Covid-19 in Netherlands and Spain.

(a). For Netherlands, based on the adjacency information of the geographic location, we can construct a regional network with 12 nodes and 20 edges. As can be seen from Figure S5.(a), a yellow warning signal was given on October 1st, which indicates that the new Covid-19 epidemic would be entering an outbreak stage. As a matter of fact, the Dutch government announced new Covid-19 prevention and control measures on September 25, lifting most of the prevention and control measures. With the relaxation of prevention and control measures, the number of new confirmed cases of

new Covid-19 in Netherlands increased rapidly, reaching an average of more than 5,000 cases per day by late October as shown in the blue curve in Figure S5.(a) and the number of new hospital admissions and severe cases also increased significantly. **(b).**For Spain, we collected the daily new Covid-19 epidemic data from May 13th in 2021 to October 12th in 2021.it can be seen from Figure S5.(b) that an early warning signal can be obtained by ARNN-LNE algorithm on June 13, indicating that Spain will soon enter the outbreak phase of the new crown epidemic in the future. Actually, despite the strong progress of the vaccination campaign, Spain began to enter a new round of the epidemic in early July. Since Spain vaccinates by age group, most young people were not vaccinated. At the end of June, the government gradually lifted some epidemic prevention measures. Frequent social activities among young people exacerbated the spread of the new Covid-19 virus. The number of new Covid-19 cases per day in Spain had been steadily rising since late June and the overall trend showed an explosive growth, reaching a peak in early August, as shown in the blue curve in Figure S5.(b).
